# 

**Published:** 1866-06

**Authors:** 


### CONTENTS FOR JUNE.

ORIGINAL CONTRIBUTIONS.
- Spinal Meningitis, or Spotted Fever. By G. C. Paoli, M. D..............p. 241
- Incised Wound of Abdomen, with Wound and Protrusion of Intestine. By A. A. Surg. R. G. Jennings, U. S. A .................................... 243
- Upon Certain Coincidences in Cases of Retained Placenta. By H. Webster Jones, A. M., M. D........................................ 244
- A Case of Placenta Prævia. By J. W. Brooks, M. D ........................ 247
- Puerperal Anasarca. By J. P. Ross, M. D.................................. 248
- Retention of the Catamenia from Obstruction. By F. C. Robinson, M.D. 252

CLINICAL REPORTS.
- A Fibrous Tumor of the Right Side of the Face. (Illustrated.) ........ 254

PROCEEDINGS OF MEDICAL SOCIETIES.
- New York Pathological Society.............................................. 255
- American Medical Association............................................... 259
- Chicago Medical Society...................................................... 279

SELECTED ARTICLES.
- *Miscellaneous*—Nitro Glycerine; Endermic Poisoning by Belladonna, etc. 284

EDITORIAL.......................................................................... 286

